# Hearing preservation after stereotactic radiosurgery for sporadic intracanalicular vestibular schwannomas classified as Koos grade 1

**DOI:** 10.1002/cam4.6990

**Published:** 2024-02-04

**Authors:** Ho Kang, So Young Ji, Chae‐Yong Kim, Ja‐Won Koo, Jae‐Jin Song, Byung Yoon Choi, Kihwan Hwang, Jung Ho Han

**Affiliations:** ^1^ Department of Neurosurgery Seoul National University Bundang Hospital, Seoul National University College of Medicine Seongnam‐si Korea; ^2^ Department of Otorhinolaryngology Seoul National University Bundang Hospital, Seoul National University College of Medicine Seongnam‐si Korea

**Keywords:** gamma knife radiosurgery, hearing preservation, radiosurgery, transient volume expansion, vestibular schwannoma

## Abstract

**Introduction:**

The mechanism of hearing loss following stereotactic radiosurgery (SRS) for vestibular schwannomas (VSs) remains unclear. There is conflicting evidence regarding cochlear nerve damage by transient volume expansion of VSs after radiosurgery and radiation‐induced cochlear damage. This study aimed to investigate whether there is a specific patient population that can achieve definite hearing preservation after SRS for VSs.

**Methods:**

A total of 37 consecutive patients with sporadic unilateral intracanalicular VSs and serviceable hearing (Gardner‐Roberson [G‐R] class I or II) were treated with SRS from 2009 to 2023. This is a retrospective study. Survival analysis with Cox regression for hearing deterioration was performed.

**Results:**

The median age was 55 years old. The median tumor volume was 0.089 cm^3^, and the median marginal dose was 12.0 Gy. Nonserviceable hearing deterioration occurred in 9 patients (24.3%), with a median onset of 11.9 months after SRS. The actuarial rates of serviceable hearing preservation were 86%, 82%, and 70% at 1, 2, and 3 years after SRS, respectively. In a multivariate analysis, only baseline pure tone average > 30 dB increased the risk of nonserviceable hearing deterioration with significant hazard ratio. There were 13 patients with petit VSs whose tumor volume was smaller than 0.05 cm^3^, and 11 of them were treated by a 4‐mm single shot with a marginal dose of 12 Gy. None of the 13 patients had nonserviceable hearing deterioration.

**Conclusions:**

Petit VSs that can be treated with 4‐mm single or double shots with a marginal dose of 12 Gy may achieve hearing preservation after SRS.

## Graphical abstract

A marginal dose >12 Gy and tumor volume >0.1 cm^3^ significantly increased the risk of nonserviceable hearing deterioration after gamma knife radiosurgery for intracanalicular vestibular schwannoma (VS). Petit VSs treated with a 4‐mm single shot with a marginal dose of 12 Gy may achieve definite hearing preservation after radiosurgery.

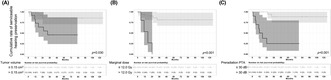

## 1 INTRODUCTION

Although the optimal treatment for vestibular schwannoma (VS) remains unclear, stereotactic radiosurgery (SRS) is widely used as a minimally invasive treatment modality.^1^, ^2^, ^3^, ^4^ Due to the increased availability of MRI, VSs, like other benign brain tumors, are often detected when they are small and asymptomatic; in such cases, the ‘wait‐and‐see’ approach may be considered, but minimally invasive SRS is also actively considered.^5^, ^6^, ^7^ However, physicians may hesitate to perform SRS due to concerns about iatrogenic hearing loss.^2^, ^3^, ^8^, ^9^, ^10^, ^11^, ^12^

The mechanism of hearing loss after SRS for VS has not yet been established, but cochlear radiation has been considered to play an important role.^2^, ^13^, ^14^, ^15^, ^16^ A cutoff mean cochlear dose of 3–5 Gy has been suggested to deteriorate hearing, and neural inflammation of the terminal of the cochlear nerve or cochlear hair cells induced by radiation has been assumed to be the biological mechanism. However, considering that the cochlear dose is determined by the size and location of the tumor, the hypothesis might also be due to a confounding effect. Another pivotal mechanism is transient volume expansion (TVE).^17^ TVE is a temporary increase in the volume of VS that mainly occurs within a year after radiosurgery. Due to the timing of TVE and hearing loss being matched, it has been suggested that spatial crowding within the internal acoustic canal (IAC) caused by TVE may cause damage to the cochlear nerve.^8^, ^18^, ^19^

In verifying hypotheses regarding the mechanism of hearing loss after SRS for VS, intracanalicular VS (IC‐VS) confined to the IAC can be a proper disease entity wherein other confounding variables are relatively well controlled because it is likely to lead to relatively less radiation exposure to the brain stem, including the cochlear nucleus, and TVE is likely to occur confined within the canal. IC‐VSs are mostly detected at small sizes, but they cannot be clinically overlooked because they occasionally grow and cause hearing loss.^18^, ^20^, ^21^, ^22^ Moreover, since IC‐VSs can be accessed through very invasive skull‐base surgical approaches, SRS is usually considered the only treatment modality. However, the risk of hearing loss after SRS still needs to be better understood.

This study aimed to analyze the hearing preservation rate after SRS for IC‐VS and to identify the risk factors for hearing loss after SRS.

## 2 MATERIALS AND METHODS

### 2.1 Study population

From September 2009 to February 2023, 37 consecutive patients with previously untreated sporadic unilateral IC‐VSs and serviceable hearing (Gardner‐Robertson [G‐R] class I or II) were treated with gamma knife radiosurgery (GKRS) at the authors' institute. The patients' medical records and images were retrospectively reviewed and analyzed. IC‐VS was defined as VS confined only to the canal corresponding to Koos grade 1, and patients diagnosed with neurofibromatosis were excluded.^23^ Among these, tumors with a volume of 0.05 cm^3^ or less that could be covered with 4‐mm single or double shots were defined as petit VSs. Audiometry and brainstem auditory evoked potential (BAEP) were performed before surgery. The result of pure‐tone audiometry was represented by a pure‐tone average (PTA), which is the arithmetic mean of the pure‐tone threshold at 500, 1000, 2000, and 4000 Hz. Then, this parameter and the result of speech discrimination score (SDS) were classified into G‐R classes. In BAEP, we focused on the interval between waves I and V, which has been reported to reflect intracanalicular pressure and hearing level.^8^ In patients where waves I or V did not form, this interval between waves I and V was replaced with the largest value measured among patients (i.e., 7.04), and statistical analysis was conducted. Tumors were also classified by location.^22^ If there was no cerebrospinal fluid (CSF) space between the cochlea and the tumor, it was defined as a fundus tumor. When the CSF space was visible on both the medial and lateral sides of the tumor, it was considered a central tumor. In a porus tumor, there is CSF space only between the cochlea and the tumor.

### 2.2 Radiosurgery and follow‐ups

Radiosurgery was performed with the Leksell Gamma Knife PERFEXION® or ICON® (Elekta, Atlanta, GA). Stereotactic MRI for target definition was performed before SRS in all cases. The marginal doses were 11.0 (*n* = 2), 11.5 (*n* = 1), 12.0 (*n* = 29), 12.5 (*n* = 1), and 13.0 Gy (*n* = 4), with a median of 12.0 Gy and an interquartile range (IQR) of 12.0–12.0 Gy. The maximal dose had a median of 24.0 Gy with an IQR of 24.0–24.3 Gy. The median number of shots was 2 with an IQR of 1–4. Acute hearing worsening was evaluated by audiometry 1 month after surgery, and the first MRI after GKRS was taken along with audiometry 3 months after surgery. Subsequently, MRI and audiometry were performed every 6 months until 2 years after GKRS and then at intervals of 1–2 years. If audiometry showed unserviceable hearing or if subjective hearing decline occurred, oral glucocorticoids were administered to promote improvement, and cases where symptoms temporarily worsened but ultimately improved to a serviceable level were classified as preserved hearing. Cochlear contouring was performed on T2‐weighted images of MRI for whole cochlea using Leksell GammaPlan® (version 11.1, Elekta, Atlanta, GA). The tumor volume was measured by summation of the areas from all sectional images using Leksell GammaPlan®. A situation where the tumor volume increased by more than 20% compared to the baseline volume and then showed a decreasing trend was defined as TVE. Tumor control was defined as a situation where the volume of the tumor does not show a continuing increasing trend and reaches a plateau or shows a decreasing trend, and additional interventions were not required due to clinical deterioration or volume increase among patients with a follow‐up of more than 1 year.

### 2.3 Statistical methods

Comparative analysis was performed between two groups according to hearing preservation or deterioration. The chi‐square test was used to compare categorical variables, and the Wilcoxon rank sum test or Student t test was conducted to compare continuous variables depending on the normality of the distribution, which was verified by the Shapiro–Wilk test. Data are presented as the median and IQR or the mean ± standard deviation, depending on the normality of the distribution. The preservation of hearing over time was quantified using Kaplan–Meier survival analysis, and risk factors for hearing deterioration were analyzed using Cox regression. The comparison between the two survival plots was conducted using the log‐rank test. In the survival analysis for patients with hearing deterioration, the period from the administration of SRS to the date of the first confirmed hearing loss in audiometry was analyzed. For patients with preserved hearing, we used the period from the SRS to the last follow‐up audiometry, or MRI, in survival analysis. In the Cox regression, tumor volume and marginal and maximal doses were divided based on the median or an approximate value from the median, and those independent variables were transformed into binary variables and analyzed. Additionally, the mean cochlear dose was made into a binary variable for analysis based on the cutoff value of 5 Gy, which was suggested as a significant cutoff for hearing loss in previous papers.^2^, ^14^, ^15^, ^16^ For multivariate Cox regression, two separate models were constructed: one model using all variables that were significant in the univariate analysis, and another model minimizing the Akaike information criterion. Multicollinearity between variables was verified using the variance inflation factor. All statistical analyses were performed using R version 4.2.3 (Foundation for Statistical Computing, Vienna, Austria).

### 2.4 Ethics statements

This study was approved by the Institutional Review Board (IRB) of the authors' institute (No. B2306‐834‐102) and conducted in compliance with the Declaration of Helsinki. Informed consent was waived by the IRB due to the retrospective study design.

## 3 RESULTS

**FIGURE 1:**
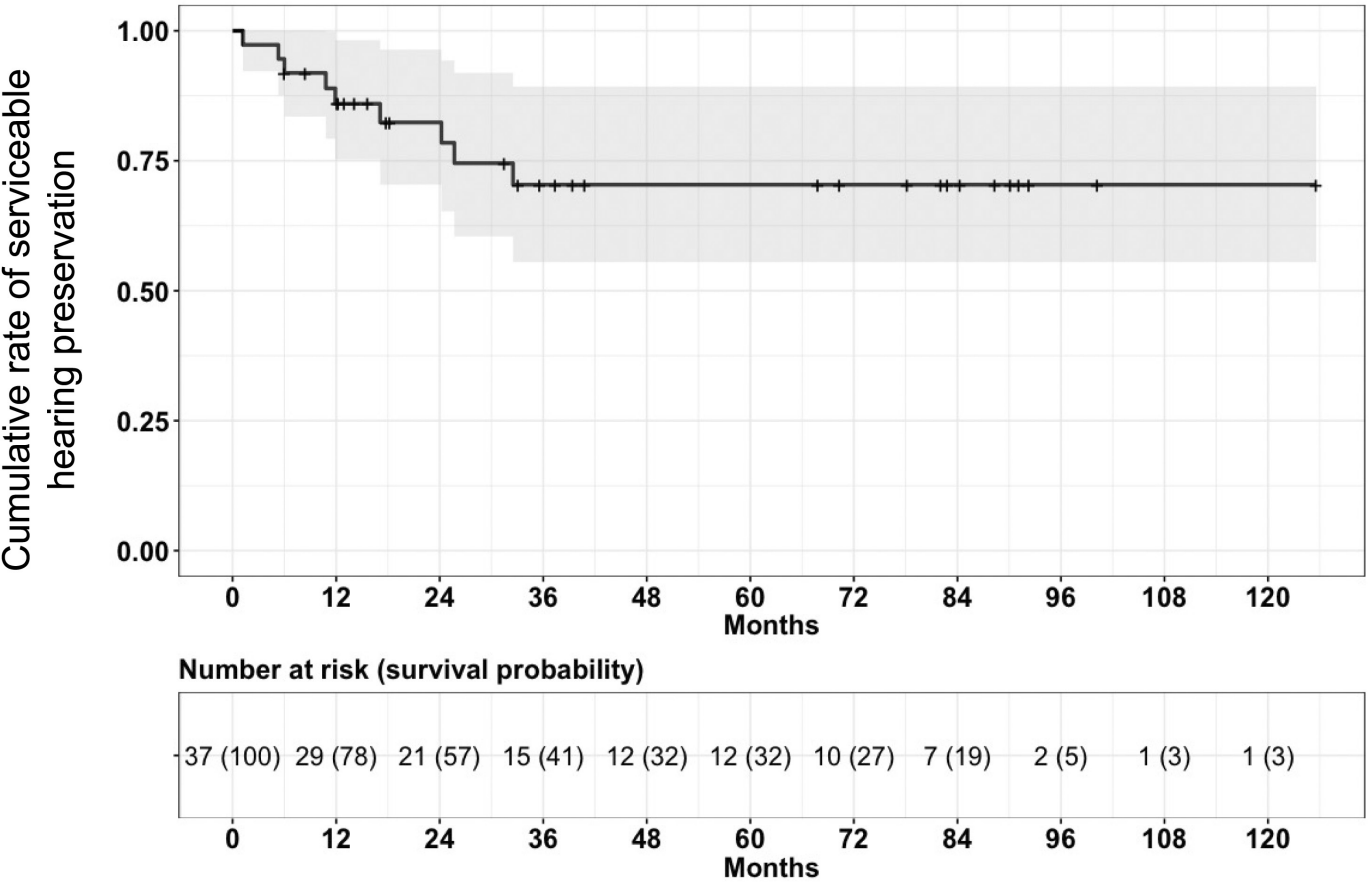
Survival plot of unserviceable hearing loss after stereotactic radiosurgery for intracanalicular vestibular schwannoma.

Baseline characteristics and circumstantial outcomes of patients according to hearing outcome are listed in Table 1. There were 9 patients (24.3%) with unserviceable hearing deterioration, which was confirmed at a median of 11.9 months (IQR, 6.0–24.2 months). The 28 patients with ultimately preserved serviceable hearing were followed up for a median of 38.4 months (IQR, 14.8–83.5 months), with a minimum follow‐up of at least 6.0 months. A median of tumor volume was 0.089 cm^3^ (IQR, 0.033–0.195 cm^3^). There were no tumors with cystic components, and there were no patients who showed facial paralysis, either temporarily or permanently. The actuarial serviceable hearing preservation rate was 86%, 82%, and 70% at 1, 2, and 3 years after SRS, respectively, and thereafter remained constant at 70% (Figure 1). There was a significant difference in mean cochlear dose according to location (fundus vs. central vs. porus, 4.6 ± 1.3 vs. 3.3 ± 1.4 vs. 2.4 ± 0.4 Gy, *p* = 0.002). In the comparative analysis, the group with worsened hearing showed significantly higher baseline PTA, lower SDS, and worse G‐R class. Additionally, the tumor volume was larger, and the marginal dose, maximal dose, and number of shots were higher in the group with worsened hearing. Excluding one patient without a post‐SRS MRI and three patients who were not followed up for more than a year, a total of 33 tumors did not grow and were controlled over a median follow‐up of 37.4 months after SRS (IQR, 15.6–84.3). Among them, 19 patients (57.6%) were followed up for over 3 years, including 16 (48.5%) for more than 5 years. Thirteen patients (36.1%) experienced transient volume expansion, which did not differ according to the preservation of hearing.

**TABLE 1:** Baseline characteristics of patients according to serviceable hearing preservation after stereotactic radiosurgery for intracanalicular vestibular schwannomas.

	Patients, No. (%)			*p*
Clinical values	All (*n* = 37)	Preserved (*n* = 28)	Deteriorated (*n* = 9)	
Age, median (IQR), y	55 (47, 59)	52 ± 9	59 ± 13	0.083
Male	16 (43.2)	14 (50.0)	2 (22.2)	0.282
Baseline pure tone average, median (IQR), dB	19.0 (11.0, 31.0)	16.6 ± 9.1	35.7 ± 13.5	**<0.001**
Speech discrimination scores, %	100 (96, 100)	100 (100, 100)	96 (96, 100)	**0.002**
Gardner‐Robertson class				**0.003**
I	28 (75.7)	25 (89.3)	3 (33.3)	
II	9 (24.3)	3 (10.7)	6 (66.7)	
Location of tumor				0.268
Fundus	14 (37.8)	9 (32.1)	5 (55.6)	0.387
Central	18 (48.6)	14 (50.0)	4 (44.4)	1.000
Porus	5 (13.5)	5 (17.9)	0 (0.0)	0.422
Interval between waves I–V in BAEP, median (IQR), ms (*n* = 31)^a^	4.32 (4.00, 4.53)	4.28 (4.00, 4.45)	5.24 (4.36, 7.04)	0.197
Tumor volume, median (IQR), cm^3^	0.089 (0.033, 0.195)	0.053 (0.031, 0.117)	0.221 (0.100, 0.263)	**0.009**
Marginal dose, median (IQR), Gy	12.0 (12.0, 12.0)	12.0 (12.0, 12.0)	12.0 (12.0, 13.0)	**0.004**
Minimal dose, median (IQR), Gy	10.1 (9.5, 11.0)	10.5 (9.7, 11.1)	10.2 (7.9,11.0)	0.534
Maximal dose, median (IQR), Gy	24.0 (24.0, 24.3)	24.0 (24.0, 24.2)	24.2 (24.1, 26.0)	**0.020**
Number of shots, median (IQR)	2 (1, 4)	2 (1, 3)	6 (3, 6)	**0.003**
Mean cochlear dose, median (IQR), Gy	3.7 (2.6, 4.2)	3.6 ± 1.4	3.9 ± 1.8	0.664
Minimal cochlear dose, median (IQR), Gy	1.7 (1.1, 2.2)	1.7 (1.2, 2.1)	2.1 (0.8, 2.6)	0.547
Maximal cochlear dose, median (IQR), Gy	7.1 (4.9, 10.1)	7.0 (4.7, 10.0)	7.7 (6.7, 10.5)	0.547
Duration until unserviceable hearing loss, median (IQR), months		–	11.9 (6.0, 24.2)	–
Follow‐up period, median (IQR), months	37.4 (15.6, 84.3)	38.4 (14.8, 83.5)	29.9 (17.1, 92.1)	1.000
Controlled tumor until last follow‐up (*n* = 33)^b^	30 (100)	23 (100)	7 (100)	–
Transient volume expansion (*n* = 36)^c^	13 (36.1)	10 (35.7)	3 (37.5)	0.926
Increase of pure tone average between baseline and last follow‐up, median (IQR), dB	6.0 (2.0, 16.0)	3.0 (0.5, 7.5)	29.0 (17.0, 35.0)	**<0.001**

*Note*: Variables were presented mean ± standard deviation or median with interquartile range according to normality.

Significant *p*‐values were annotated as **BOLD**.

Abbreviations: BAEP, brainstem auditory evoked potentials; IQR, interquartile range.

^a^ Preoperative BAEP was performed only in 31 patients.

^b^ A total of 4 patients, one patient without follow‐up MRI and 3 patients who were not followed up for more than 1 year, were excluded from the analysis.

^c^ One patient without follow‐up MRI was excluded from the analysis.

**FIGURE 2:**
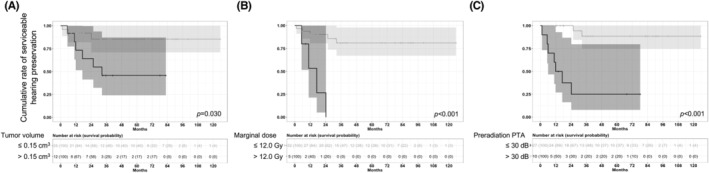
Survival plots for serviceable hearing preservation with 95% confidence intervals according to (A) tumor volume >0.15 cm^3^, (B) marginal dose >12 Gy, and (C) Gardner‐Robertson class. *p*‐Values were analyzed using log‐rank test.

We presented hazard ratios and survival plot according to selected variables in Table 2 and Figure 2, respectively. In the univariate analysis, the risk of hearing deterioration increased when the tumor volume exceeded 0.15 cm^3^ (hazard ratio [HR], 4.1; *p* = 0.045), the marginal dose exceeded 12.0 Gy (HR, 13.2; *p* < 0.001), preoperative G‐R class II (HR, 9.6; *p* = 0.002), and preoperative PTA exceeded 30 dB (HR, 16.6; *p* < 0.001) were present. In the two different multivariate model using these significant variables, only preoperative PTA higher than 30 dB significantly increased the risk of hearing deterioration with HRs of 9.6 (*p* = 0.014) and 11.2 (*p* = 0.008), respectively.

**TABLE 2:** Cox regression model for the risk of unserviceable hearing loss after stereotactic radiosurgery for intracanalicular vestibular schwannoma for selected variables.

	*n*	Univariate		Multivariate (Model 1)^a^		Multivariate (Model 2)^b^	
Variables		HR (95% CI)	*p*	HR (95% CI)	*p*	HR (95% CI)	*p*
Female	21	3.1 (0.6–15.0)	0.167	–	–	–	–
Age >60 years	7	3.3 (0.8–13.3)	0.097	–	–	–	–
Tumor volume >0.15 cm^3^	12	4.1 (1.0–16.5)	**0.045**	2.4 (0.6–10.0)	0.235	–	–
Marginal dose >12.0 Gy	5	13.2 (2.9–60.8)	**<0.001**	2.9 (0.6–15.6)	0.203	3.1 (0.6–15.9)	0.172
Maximal dose >24.0 Gy^c^	15	4.7 (1.0–22.5)	0.055	–	–	–	–
Mean cochlear dose >5.0 Gy	8	1.1 (0.2–5.4)	0.885	–	–	–	–
Preradiation G‐R class II	9	9.6 (2.3–39.2)	**0.002**			–	–
Preradiation PTA >30 dB	10	16.6 (3.4–81.9)	**<0.001**	9.6 (1.6–57.8)	**0.014**	11.2 (1.9–65.7)	**0.008**
Fundus tumor	14	1.7 (0.5–6.4)	0.423	–	–	–	–

Abbreviations: CI, confidence interval; G‐R class, Gardner‐Robertson class; HR, hazard ratio; PTA, Pure‐tone average.

Significant *p*‐values were annotated as **BOLD**.

^a^ Model 1 was analyzed using all significant variables identified in the univariate analysis. Preradiation G‐R class was excluded in Model 1 because of the collinearity problem.

^b^ Model 2 was analyzed using the combination of significant variables from the univariate analysis that demonstrated the lowest Akaike information criterion value.

^c^ This variable was excluded in the multivariate analysis due to multicollinearity.

**FIGURE 3:**
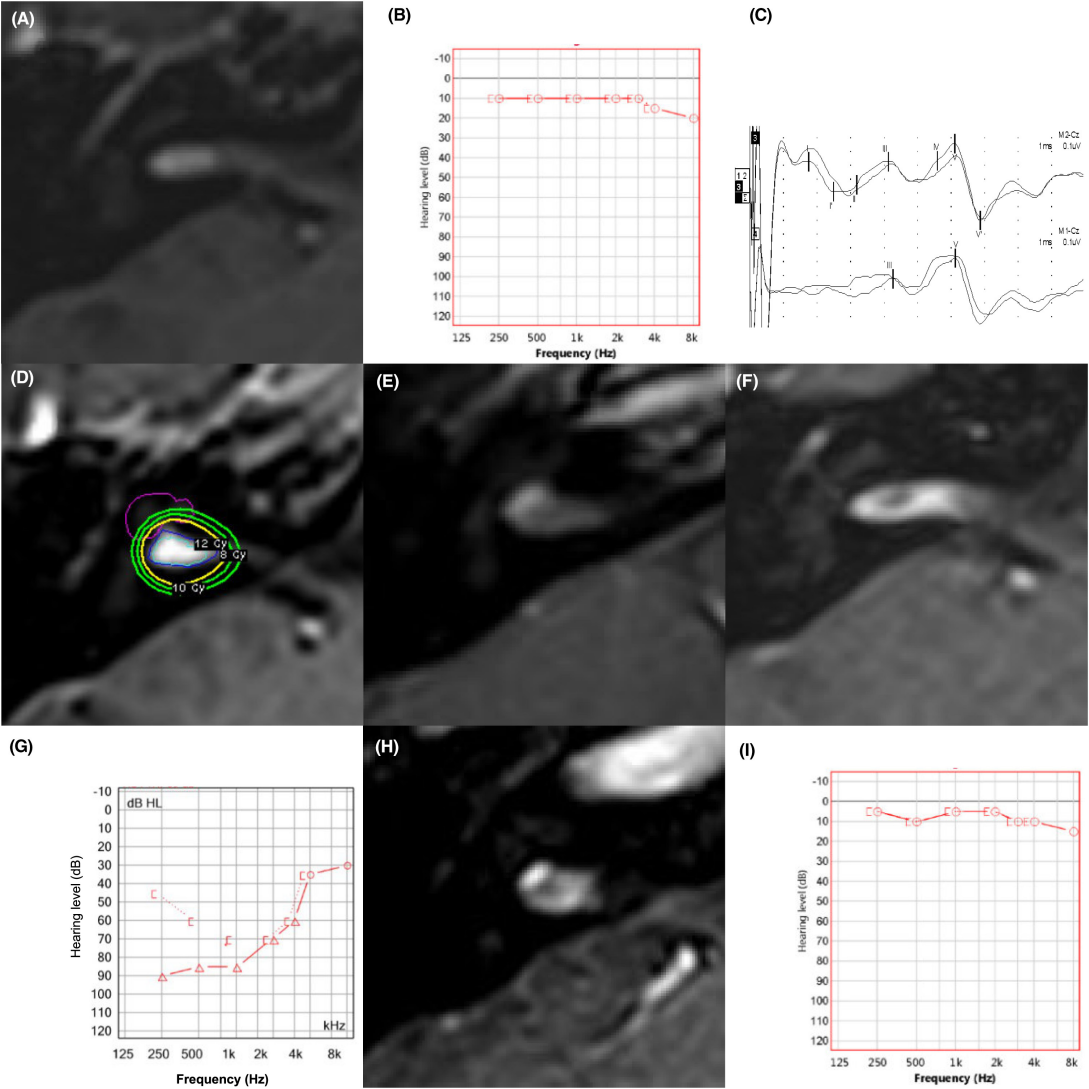
A representative case of a petit vestibular schwannoma. A 38‐year‐old female patient with a petit vestibular schwannoma on the right side with a volume of 0.045 cm^3^ (A). She had serviceable hearing with an average of 11 dB in pure tone audiometry (B), and in the preradiation brain stem auditory evoked potential, the difference in latency between waves I and V was 4.36 ms on the right side (C). Gamma knife radiosurgery was performed using a 4‐mm dual shot with a marginal dose of 12 Gy (D). Six days after radiosurgery, the tumor showed the minimal change in MRI taken due to neck discomfort (E). The volume of the tumor increased to 0.056 cm^3^ at 6 months after radiosurgery (F), and hearing was deteriorated to the unserviceable range with a PTA of 75 dB (G). Thirty‐one months after radiosurgery, the volume of the tumor decreased (H), and the average PTA improved to 7 dB (I).

There were 13 patients (35.1%) with petit VSs, with a median age of 54 (IQR, 47–57 years old) and a median tumor volume of 0.030 cm^3^ (IQR, 0.013–0.033 cm^3^). Among them, 4 patients (30.8%) had fundus tumors, and the others (9 patients, 69.2%) had central tumors. The median preoperative PTA was 13 dB (IQR, 11–19 dB), with only a single patient being G‐R class II. GKRS was performed with a 4‐mm single shot in 11 patients, while 2 patients required a 4‐mm double shot. The median of the mean cochlear dose was 4.0 Gy (IQR, 2.9–5.1 Gy). The petit schwannomas were followed up for a median of 37.4 months (IQR, 15.6–88.3 months), and all patients preserved their serviceable hearing. A representative case of a petit schwannoma is presented in Figure 3. For a fundus‐type IC‐VS with a tumor volume of 0.045 cm^3^, GKRS was performed with a marginal dose of 12 Gy with double shots. She had G‐R class I baseline hearing function with a PTA of 11 dB. Six months after GKRS, TVE appeared, and the PTA increased to 75 dB. Oral steroids were administered for 3 weeks. At 31 months after GKRS, the tumor volume decreased, and the PTA improved to 7 dB.

## 4 DISCUSSION

In this series of 37 patients with IC‐VS with serviceable hearing who underwent SRS, the smaller the tumor, the lower the marginal dose, and the better the preoperative hearing function, the more serviceable hearing was preserved after SRS. Tumor control was achieved in all patients with a median marginal dose of 12 Gy. In particular, for petit VSs with a tumor volume of 0.05 cm^3^ or less, serviceable hearing was preserved in all patients.

There is still debate about the optimal marginal dose that can achieve tumor control while promoting hearing preservation in VS, but most previous studies suggested 12–13 Gy.^2^, ^5^, ^6^, ^9^, ^13^, ^14^, ^16^, ^24^ In this study, the hearing preservation rate significantly decreased when a dose exceeding 12.0 Gy was administered, and it is difficult to determine whether the difference in cochlear dose is the cause of hearing deterioration because there was no difference in hearing preservation according to the cochlear dose. In the correlation analysis among variables, only baseline hearing function was significantly correlated with marginal dose (Figure S1). This implies that patients with a baseline hearing of G‐R class II were irradiated with a dose 0.5–1.0 Gy higher than those with a baseline hearing of G‐R class I to prioritize assured tumor control with a relatively higher anticipated risk of serviceable hearing loss. This is based on the understanding that administering a higher dose increases the possibility of achieving better tumor control.^25^ Consequently, the difference in hearing outcomes based on the marginal dose was due to baseline hearing function as a confounding variable.

The key point of this study is that for SRS on IC‐VS, the cochlear dose was not a risk factor for hearing loss, and the volume of the tumor was one of the critical risk factors. Given that nearly half of this study included cases where the cochlear dose was higher than the risky cutoff cochlear dose of 3–5 Gy suggested in previous studies, if the cochlear dose were a major cause of hearing loss, this study should also have shown a significant difference in hearing preservation rates according to this. In this series, since the marginal dose was only given within the range of 11.0–13.0 Gy, the cochlear dose ultimately depends on how close the tumor is located to the cochlear fundus; indeed, there was a significant difference in mean cochlear dose according to location. In this study, neither cochlear dose nor its location affected hearing preservation. On the other hand, the most pivotal characteristic of the tumor that significantly affected hearing in our study was the volume of the tumor, which implies a mechanism of hearing loss due to TVE, as crowding in the IAC during or after TVE would be more severe when the tumor is larger.^18^, ^19^ However, in this study, the presence or absence of TVE did not show significant differences according to hearing loss; there is a possibility that TVE was not detected at our MRI follow‐up points. The timing and duration of TVE have not yet been established, and since VS is a benign disease, it is difficult to justify taking MRIs too often.

Despite 100% preservation of serviceable hearing after SRS for petit VS in this study, whether SRS is necessary for such small IC‐VSs remains unclear. This is because we do not yet fully understand the natural course of petit VS. However, some previous studies reported that untreated IC‐VSs occasionally grow or cause hearing loss, which could serve as a basis for proactive SRS.^22^, ^26^ Also, a recent randomized controlled study supported that upfront radiosurgery is more effective in reducing volume of the tumor compared to the wait‐and‐scan approach in small‐ or medium‐sized VS.^24^ In this study, SRS for petit VS was not entirely without hearing deterioration. Although the PTA only worsened by a median of 2 dB after SRS compared with baseline, it worsened by more than 15 dB in two patients, shifting from G‐R class I to II. These two patients had the largest volume among the petit VS group (Figure S2). As patients with petit VS mostly have G‐R class I hearing and the hearing preservation rate after SRS with preoperative G‐R class II hearing is significantly lower.^27^ We could consider a treatment strategy of performing proactive SRS for petit VS before it grows to become G‐R class II.

This study has several limitations. The number of petit VS cases was only 13, so it is difficult to conclude that SRS for petit VS is safe based solely on this study. However, based on the results of this study, there is no need to avoid SRS for petit VS due to excessive worry about the risks of cochlear dose, as suggested in previous studies. The retrospective design and the fact that the study was from a single institution are also limitations.

## 5 CONCLUSIONS

A large tumor volume, a marginal dose exceeding 12.0 Gy, and worsened preoperative hearing function were risk factors for unserviceable hearing deterioration after SRS in IC‐VSs with serviceable hearing. Petit VSs that can be treated with 4‐mm single or double shots with a marginal dose of 12 Gy may achieve hearing preservation after SRS.

## AUTHOR CONTRIBUTIONS

**Ho Kang:** Data curation (lead); formal analysis (lead); writing – original draft (lead); writing – review and editing (lead). **So Young Ji:** Data curation (supporting); investigation (supporting); validation (lead); funding acquisition (lead). **Chae‐Yong Kim:** Resources (supporting); supervision (supporting). **Ja‐Won Koo:** Data curation (supporting); resources (supporting); supervision (supporting); validation (supporting). **Jae‐Jin Song:** Data curation (supporting); resources (supporting); validation (supporting). **Byung Yoon Choi:** Data curation (supporting); resources (supporting); validation (supporting). **Kihwan Hwang:** Data curation (supporting); investigation (supporting); resources (supporting); validation (supporting). **Jung Ho Han:** Conceptualization (lead); data curation (lead); methodology (lead); resources (lead); supervision (lead), funding acquisition (supporting).

## CONFLICT OF INTEREST STATEMENT

The authors have no conflicts of interest to declare.

## Supporting information


Figure S1‐S2.

## Data Availability

The institutional data are not publicly available due to the protection of private patient health information. However, the datasets generated during and/or analyzed during the current study are available after anonymization from the corresponding author on reasonable request.
