# Supplementary material for: Hearing preservation after stereotactic radiosurgery for sporadic intracanalicular vestibular schwannomas classified as Koos grade 1
Source: Cancer Med. 2024 Feb 4;13(2):e6990. doi: 10.1002/cam4.6990 (PMC10839155; doi:10.1002/cam4.6990)

Supplementary Figure S1. Correlation plot with selected variables

Pearson correlation coefficients were presented with asterisks according to p-values.
*, p<0.05; **, p<0.01; ***, p<0.001

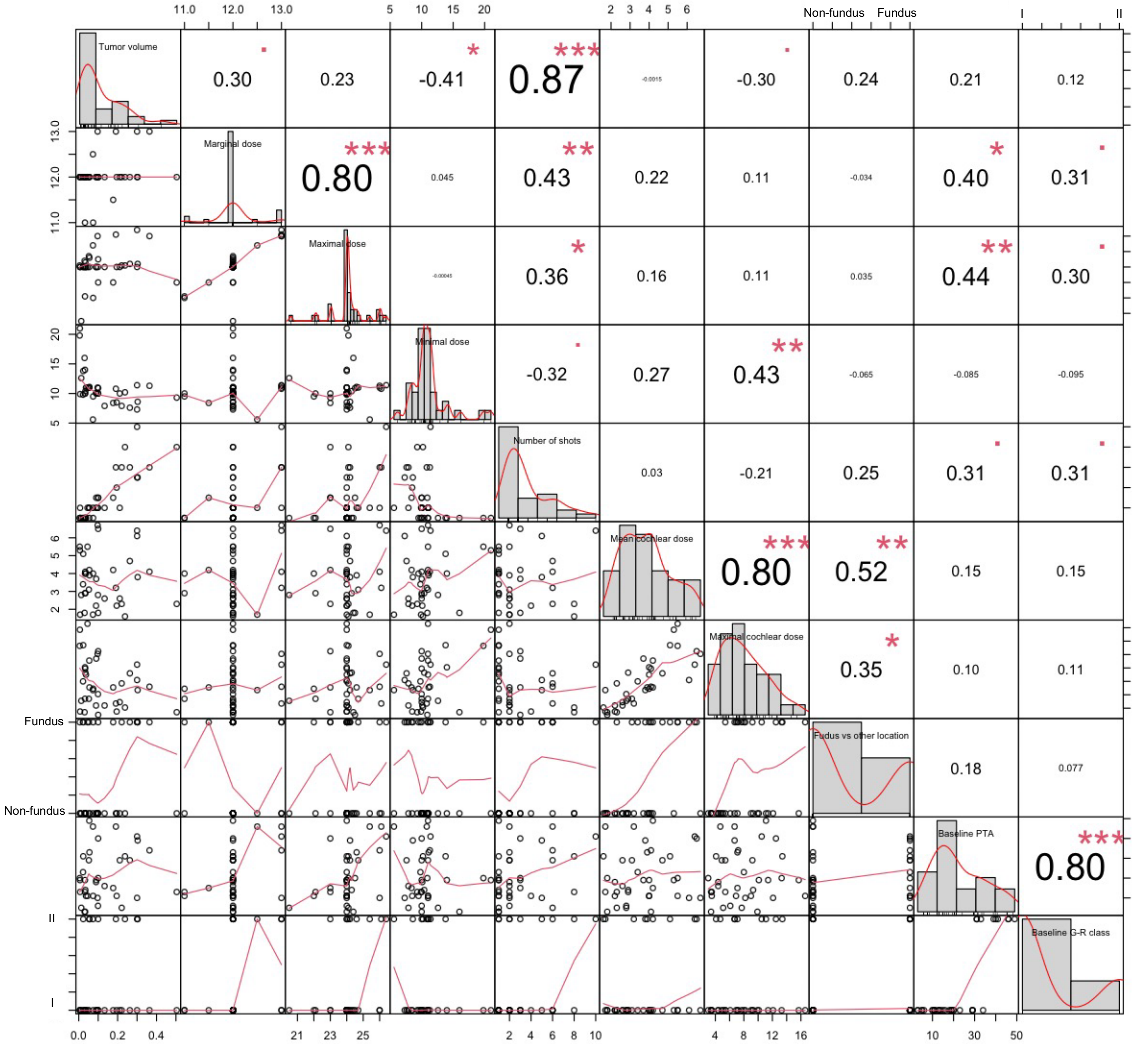

Supplementary Figure S2. Changes of pure tone average between before radiosurgery and last follow up among overall patients (A) and petit vestibular schwannomas (B)

Patients with an increase in pure tone average of 10 dB or more were indicated in red.

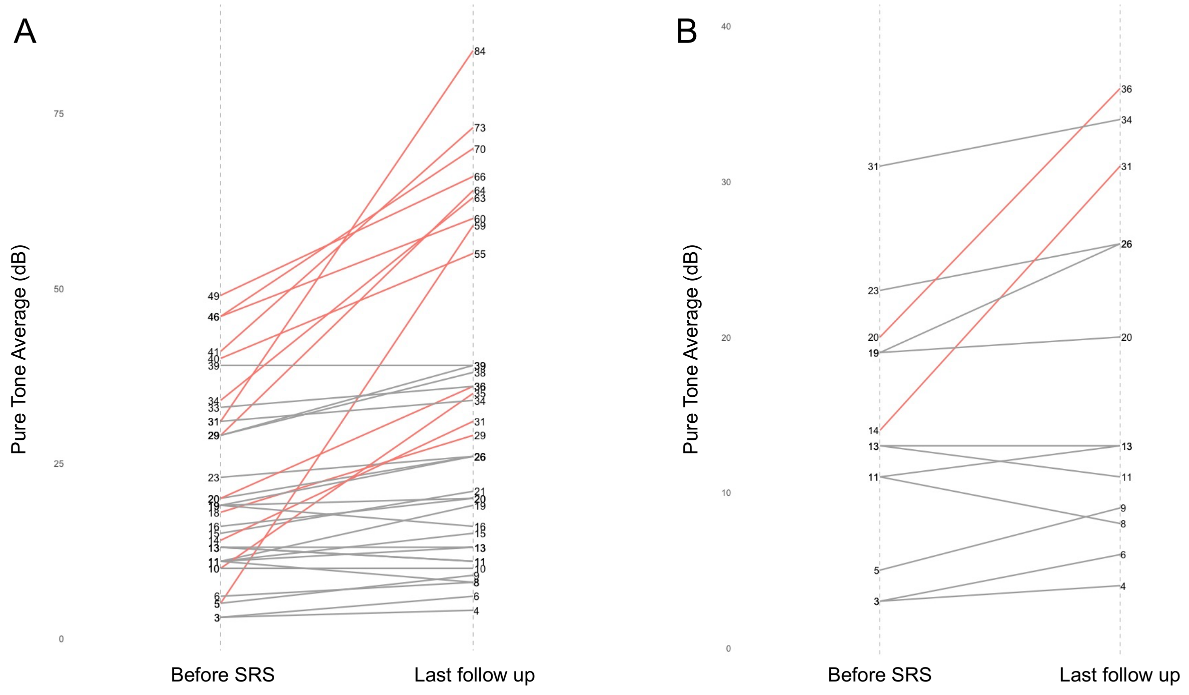

Supplement: Supplementary file 1 — Figure S1‐S2. [file CAM4-13-e6990-s001.pdf]
